# Abnormal brain functional network dynamics in obsessive–compulsive disorder patients and their unaffected first‐degree relatives

**DOI:** 10.1002/hbm.25555

**Published:** 2021-06-05

**Authors:** Ziwen Peng, Ya Guo, Xiangshu Wu, Qiong Yang, Zhen Wei, Carol A. Seger, Qi Chen

**Affiliations:** ^1^ Key Laboratory of Brain, Cognition and Education Sciences, Ministry of Education, China; School of Psychology, Center for Studies of Psychological Application, and Guangdong Key Laboratory of Mental Health and Cognitive Science South China Normal University Guangzhou China; ^2^ Department of Psychiatry Southern Medical University Guangzhou China; ^3^ Affiliated Brain Hospital of Guangzhou Medical University Guangzhou China; ^4^ Department of Child Psychiatry and Rehabilitation Affiliated Shenzhen Maternity & Child Healthcare Hospital, Southern Medical University Shenzhen China; ^5^ Department of Psychology Colorado State University Fort Collins Colorado USA

**Keywords:** dynamic functional network connectivity, endophenotype, first‐degree relatives, functional magnetic resonance imaging, obsessive–compulsive disorder

## Abstract

We utilized dynamic functional network connectivity (dFNC) analysis to compare participants with obsessive–compulsive disorder (OCD) with their unaffected first‐degree relative (UFDR) and healthy controls (HC). Resting state fMRI was performed on 46 OCD, 24 UFDR, and 49 HCs, along with clinical assessments. dFNC analyses revealed two distinct connectivity states: a less frequent, integrated state characterized by the predominance of between‐network connections (State I), and a more frequent, segregated state with strong within‐network connections (State II). OCD patients spent more time in State II and less time in State I than HC, as measured by fractional windows and mean dwell time. Time in each state for the UFDR were intermediate between OCD patients and HC. Within the OCD group, fractional windows of time spent in State I was positively correlated with OCD symptoms (as measured by the obsessive compulsive inventory‐revised [OCI‐R], *r* = .343, *p*<.05, FDR correction) and time in State II was negatively correlated with symptoms (*r* = −.343, *p*<.05, FDR correction). Within each state we also examined connectivity within and between established intrinsic connectivity networks, and found that UFDR were similar to the OCD group in State I, but more similar to the HC groups in State II. The similarities between OCD and UFDR groups in temporal properties and State I connectivity indicate that these features may reflect the endophenotype for OCD. These results indicate that the temporal dynamics of functional connectivity could be a useful biomarker to identify those at risk.

## Graphical abstract

We identified several dFNC differences in OCD and UFDR that may be useful for establishing biomarkers of an OCD endophenotype. First, OCD patients had abnormal temporal properties which correlated with clinical features, which were shared with their UFDR. Second, we found similar connectivity patterns for UFDR and OCD within a dFNC state characterized by between‐network integration. These results provide new insights into the pathophysiology of OCD patients and indicate dynamic functional connectivity measures that could be used as biomarker to identify those at risk.

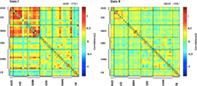

## 1 INTRODUCTION

Obsessive–compulsive disorder (OCD) is a group of neuropsychiatric diseases with obsessive thinking and compulsive behavior as the main clinical manifestations. OCD is common in the general population (2.5–3%) (Robbins, Vaghi, & Banca, 2019). Family members of OCD patients are at a higher risk for OCD compared with the general population (Nestadt, Grados, & Samuels, 2010), indicating a high genetic risk.

In recent years, the development of neuroimaging technology has provided a new way to explore the pathophysiological mechanisms underlying obsessive–compulsive disorder. Particularly, resting‐state functional magnetic resonance imaging (rs‐fMRI) has attracted attention due to a number of advantages: it is noninvasive, easy to perform, can be repeated, and avoids individual differences in the execution of tasks that might complicated the use of task‐based fMRI (Barkhof, Haller, & Rombouts, 2014). Numerous rs‐fMRI studies have identified abnormalities of the cortico‐striato‐thalamo‐cortical circuit (CSTC) as a common characteristic in OCD patients (Calzà et al., 2019; Jung et al., 2013; Posner et al., 2014; van den Heuvel et al., 2016; Zhao et al., 2019). However, previous neuroimaging studies have suggested that abnormalities are not limited to the CSTC circuit and other regions (Anticevic et al., 2014; De Wit et al., 2014; Hou et al., 2014; Milad et al., 2013), but also can be seen in cortical brain network connectivity (Fan et al., 2017a; Gürsel, Avram, Sorg, Brandl, & Koch, 2018; Shin et al., 2014).

Most previous rs‐fMRI studies on OCD patients have investigated FC patterns as a static phenomenon. Recent studies have found that FC varies over time (Calhoun, Miller, Pearlson, & Adali, 2014) and such temporal fluctuations can be captured by dynamic functional network connectivity (dFNC) methods, providing greater insight into the fundamental properties of brain networks (Hutchison et al., 2013). Studies of a variety of psychiatric disorders have revealed that abnormal dFNC characteristics (Espinoza et al., 2019), including in autism spectrum disorder (ASD) (de Lacy, Doherty, King, Rachakonda, & Calhoun, 2017), schizophrenia (Rabany et al., 2019), and major depression (Han et al., 2020). Previous dFNC studies of OCD patients have been limited in many ways. Gürsel and colleagues performed group‐based independent component and sliding time window analyses to investigate dFNC alterations (Gürsel et al., 2020). They focused on a subset of networks (default mode network, frontoparietal network, and salience network) and did not examine whole brain connectivity patterns. Liu and colleagues examined first‐episode and treatment‐naive patients with obsessive–compulsive disorder (OCD) (Liu et al., 2020) and did not examine patients undergoing treatment. Neither study examined nonaffected relatives in order to examine whether dFNC patterns might serve to identify endophenotypes for OCD.

Endophenotypes have been defined as “measurable components unseen by the unaided eye along the pathway between disease and distal genotype” (Gottesman & Gould, 2003). Endophenotypes can serve as a more direct indicator of a genetic component of a disease than overt disease symptoms. Endophenotypes are often present in unaffected family members at a higher rate than in the general population. Therefore, data from unaffected relatives of those with OCD is critical for identifying common endophenotypes that can be used for diagnosis and treatment. Relatives of OCD patients are more likely to suffer from OCD than the general population (Gottesman & Gould, 2003; Pauls, 2008; Pauls, Abramovitch, Rauch, & Geller, 2014). The concept of endophenotype (Gottesman & Shields, 1973) has proven useful in helping to bridge the gap between genetics and behavioral disease processes and has been widely used in the study of psychiatric illnesses including OCD, schizophrenia, attention deficit hyperactivity disorder (ADHD), and depression (Chamberlain et al., 2008; Chamberlain & Menzies, 2009; De Vries et al., 2014; Gottesman & Gould, 2003; Gould & Gottesman, 2006; Menzies et al., 2007; Peng et al., 2014; Peng et al., 2014; Peng et al., 2015; Shaw et al., 2015; Viswanath, Janardhan Reddy, Kumar, Kandavel, & Chandrashekar, 2009). The clinical relevance and potential biomarker utility of dFNC in particular is supported by clinical studies of schizophrenia (Du et al., 2016), autism (Yao et al., 2016) and Parkinson disease (Kim et al., 2017). A primary goal of our study was to examine whether dFNC properties might serve as biomarker for OCD by directly comparing OCD patients with their unaffected first‐degree relatives as well as healthy controls with no family history of OCD.

We performed group ICA on rs‐fMRI and a sliding‐window analysis to compare dFNC in OCD patients, their unaffected first‐degree relatives (UFDR) and healthy control participants (HC). We hypothesized that (a) OCD patients would show altered dFNC, compared with healthy controls. (b) Clinical features in OCD would correlate with altered dFNC temporal properties and (c) UFDR may show dFNC disruption similar to that found in OCD.

## 2 MATERIALS AND METHODS

### 2.1 Participants

We enrolled 48 OCD patients, 24 UFDR, and 49 HC. All participants gave informed consent according to the institutional research and ethics committee of the Guangzhou Psychiatric Hospital. The groups were matched on age (range between 18 and 50) and gender. All participants were right‐handed. OCD patients and their UFDR were recruited from the Guangzhou Psychiatric Hospital. HC were recruited through local and community advertisements. In order to maintain diagnostic consistency over time within our lab's OCD database, all patients were diagnosed according to DSM‐IV criteria using the Structured Clinical Interview (SCID) for DSM‐IV‐TR Axis I disorders. All participants were diagnosed by one experienced clinical psychiatrist and one experienced psychologist. All subjects gave written informed consent before participation.

Patients were excluded: (a) if they had a history of brain trauma or neurological disease; (b) if they had a history of alcohol or substance abuse. Twenty‐six OCD patients were receiving treatment with selective serotonin reuptake inhibitors; all had been stable on their medication for at least 4 weeks. Details of the medications and dosages for OCD patients are provided in the supplementary materials (Table S1). Twenty‐five patients had comorbidity with anxiety symptoms, and eight had depression symptoms. Comorbid anxious and depressive symptoms were not considered as an exclusion criterion, provided that OCD was the primary clinical diagnosis.

The exclusion criteria for UFDR and HC were same as those for the OCD patients. In addition, they were excluded if they reported any history of mental illness and/or treatment with any psychotropic medication as screened by using the SCID for DSM‐IV‐TR AXIS I disorders. If HC had a family history of any psychiatric disorders as defined by the DSM‐IV, they were also excluded.

### 2.2 Clinical assessments

The Yale‐Brown obsessive–compulsive scale (Y‐BOCS) was administered to assess illness severity (Goodman et al., 1989). The obsessive–compulsive inventory‐revised (OCI‐R) (First, Spitzer, & Gibbon, 2002; Peng, Yang, Miao, Jing, & Chan, 2011) was used to identify categories of OCD symptoms including obsession, washing, checking, neutralizing, ordering, and hoarding. The Beck Depression Inventory (BDI) (Beck & Steer, 1984) was used to assess depressive symptoms, and the State–Trait Anxiety Inventory (STAI) Y‐1 and Y‐2 (Spielberger, Gorsuch, & Lushene, 1970) to assess anxiety symptoms. Analysis of variance (ANOVA) and Chi‐square tests were used to test for group differences in these variables using SPSS 22.0 for Windows.

### 2.3 Data acquisition and preprocessing

An Achieva 3.0‐Tesla MR system (Philips Medical System, Amsterdam, The Netherlands.) equipped with an eight‐channel phased‐array head coil was used for data acquisition. Functional data were collected using gradient echo Echo‐Planar Imaging (EPI) sequences (time repetition, TR = 2000 ms; echo time, TE = 30 ms; flip angle = 90°, 33 slices, field of view [FOV] = 220 mm × 220 mm, matrix = 64 × 64; slice thickness = 4.0 mm; voxel size =3.4 × 3.4 × 4 mm^3^). For each participant, the fMRI scanning lasted for 480 s, and 240 volumes were obtained. During the scanning, participants were instructed to relax with eyes closed, and stay awake without moving. For spatial normalization and localization, a high‐resolution T1‐weighted anatomical image was also acquired using a magnetization prepared gradient echo sequence (TR = 8 ms, TE = 3.7 ms, inversion time = 0, flip angle = 7°, FOV = 240 mm × 240 mm, matrix =256 × 256, slice thickness = 1.0 mm; voxel size =1.0 × 1.0 × 1.0 mm^3^).

Data preprocessing was carried out using the Statistical Parametric Mapping toolbox (SPM12, https://www.fil.ion.ucl.ac.uk/spm), and Data Processing Assistant for Resting‐State fMRI (DPARSFA version 4.4, http://rfmri.org/dpabi). Image preprocessing consisted of: (a) slice timing correction; (b) motion correction in which the functional images in the BOLD sequence were realigned to the first volume; (c) spatial normalization into the stereotactic space of the Montreal Neurological Institute and resampling at 3 × 3 × 3 mm^3^; (d) spatial smoothing with a 8‐mm full‐width at 2/3–12 maximum isotropic Gaussian kernel. After preprocessing, two OCD patients were excluded due to head motion greater than 2 mm or 2°.

### 2.4 Group independent component analysis

**FIGURE 1:**
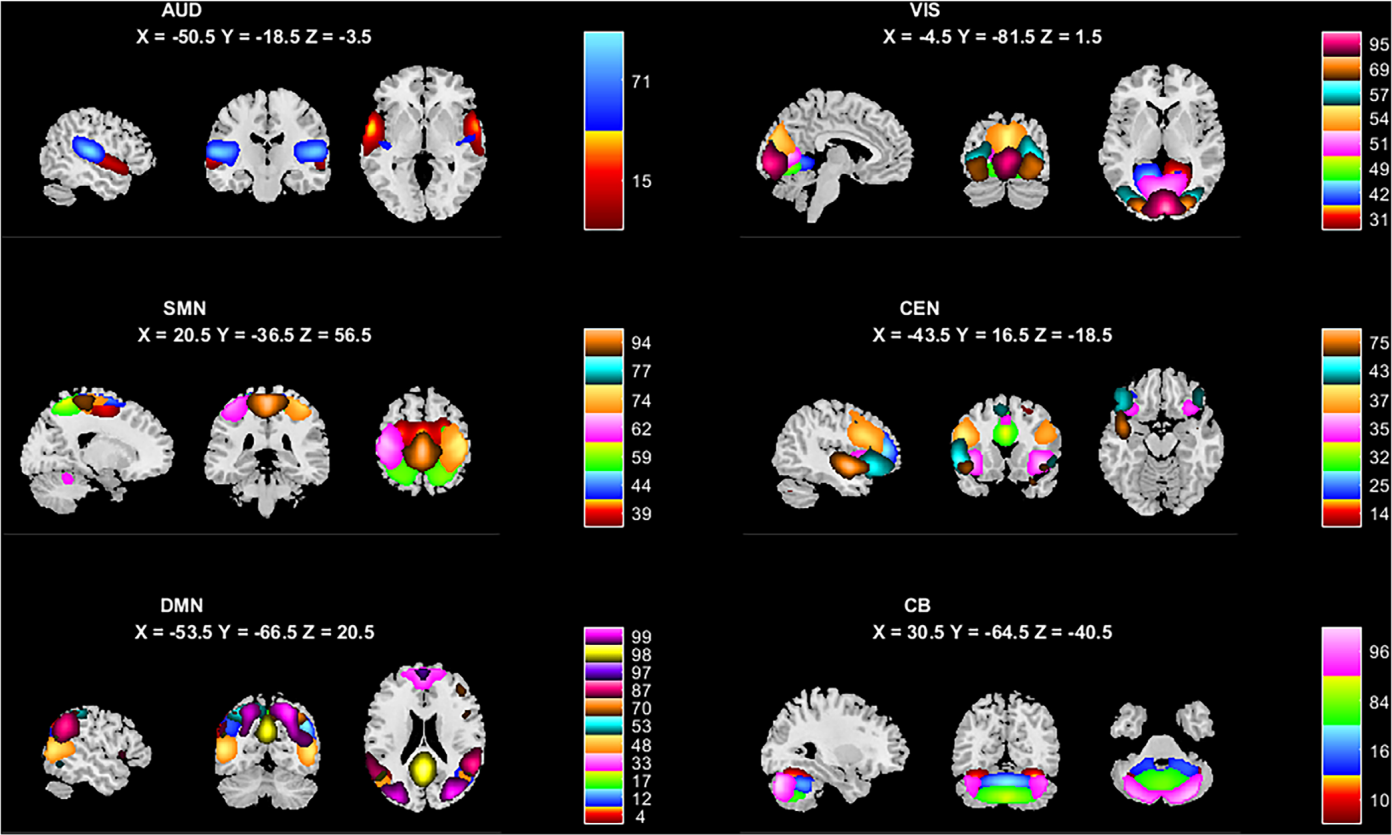
Spatial maps of the 39 independent components (ICs) divided into six networks. Auditory network (AUD); visual network (VIS); sensorimotor network (SMN); cognitive executive network (CEN); default mode network (DMN); cerebellar (CB) network. Different colors represent specific ICs

After data preprocessing, the resting state data was analyzed using spatial group independent component analysis (sGICA) as implemented in the GIFT software toolbox (GIFT v4.0a; http://icatb.sourceforge.net). Specific steps were as follows: (a) We used principal component analysis (PCA) to reduce the dimensionality of the data in two steps. We set the number of independent components to 100 in advance, process the data of single participants individually, and then connect all the participants' data into groups for processing. (b) The Infomax algorithm was used to estimate the independent components. It was repeated 100 times in ICASSO (Bell & Sejnowski, 1995) to ensure the stability of the results. (c) The independent components (including spatial maps and time series) for each subject were reconstructed in reverse and Fisher Z transformation was performed. The data after Z transformation approximately obeyed the normal distribution with the mean value of the *SD*. (d) The Display GUI module in the GIFT toolbox was used to identified relevant network components. To identify which of the 100 ICs were meaningful, we chose those for which the peak activation coordinates were located primarily in gray matter and had low levels of spatial overlap with vascular, ventricular corresponding to artifacts (Allen et al., 2011). We further used Stanford functional ROIs (http://findlab.stanford.edu/functional_ROIs.html) as templates to select ICs with high similarity to the templates. All ICs retained for analysis were located on gray matter, had low spatial overlap with cerebral ventricles and blood vessels, and had time courses dominated by low frequency signals (ratio of powers below 0.1 Hz to 0.15–0.25 Hz in spectrum) (Allen et al., 2014). A total of 39 meaningful ICs were identified according to these criteria. These ICs fell within the following functional networks: auditory (AUD), visual (VIS), sensorimotor (SMN), cognitive executive (CEN), default mode (DMN), and cerebellar (CB) networks (Figure 1 and Table S2).

Additional postprocessing was applied to the time courses of the 39 meaningful independent components to remove remaining noise sources (Allen et al., 2014). Subject specific time courses were detrended and despiked using 3dDespike, then filtered using a fifth‐order Butterworth low‐pass filter with a high frequency cutoff of 0.15 Hz.

### 2.5 dFNC analysis

The GIFT toolbox was applied to calculate dFNC through a sliding window analysis followed by k‐means clustering. First, we used a sliding‐window approach, in which a sliding time window of the 22‐repetition time (TRs) method was applied to each participant, with a Gaussian window alpha value of 3 and a step between windows of 1 TR, resulting in 208 consecutive windows. We also analyzed the effect of different window lengths on the results. The results were highly consistent across a wide range of window sizes (18–26 TRs), suggesting that the identified altered dFNC was not caused by random artifacts related to window size (Figures S1–S4). To promote sparsity in the estimations, a penalty was imposed on the L1 norm of the precision matrix. We then applied a k‐means clustering algorithm to the resulting 208 FC window FNC matrices for all the participants, which was iterated 150 times. The dFNC matrices of all participants were then clustered by using the k‐means algorithm to assess the frequency and structure of the recurring FNC patterns. There are several different rules of thumb for determining the appropriate value of cluster number K (Allen et al., 2014; Yang et al., 2021). In this study, we used the elbow criterion of the cluster validity index, and the optimal number of clusters was set as 2, which we refer to as State I and State II.

### 2.6 Group differences in dynamic connectivity: Temporal properties and strength

We used three different indices to examine the temporal properties of the dFNC states: (a) Fractional windows (FW) defined as the proportion of time spent in each state; (b) mean dwell time (DT) defined as the mean length of time the participant remained in each state before switching to another state (c) number of transitions, defined as the total number of changes between states the participant made across the entire resting state scan. Group differences were assessed using ANOVA, applying a least significant difference post hoc test. ANOVA was also used to compare the connectivity strength between individual pairs of ICs pairing within each state (*p*< .05, false discovery rate (FDR) correction (Benjamini & Hochberg, 1995) between the three groups, applying a least significant difference post hoc test. The total number of connections taken into account by the multiple comparisons correction was 741.

### 2.7 Clinical and neuropsychological data analysis

Statistical analyses were performed using SPSS 22.0 for Windows. One‐way ANOVA was used to test for between‐group differences (OCD, UFDR, and HC). Chi‐square tests were used for categorical variables. Within the OCD group we performed correlation analyses between altered temporal properties and obsessional symptom scores [Yale‐Brown obsessive–compulsive scale (YBOC‐S), and obsessive compulsive inventory‐revised (OCI‐R)], controlling for depression (BDI scores), anxiety (STAI scores), and education level. Statistical significance threshold was set at *p*<.05, FDR correction.

## 3 RESULTS

### 3.1 Demographic and clinical characteristics

No significant differences were found among OCD, UFDR and HC in age (F _[2,116]_ = 1.574, *p* = .212) and gender (χ^2^ = 4.136, *p* = .126) (Table 1). Years of education for the HC group was higher than the other two groups (F _[2,116]_ = 3.942, *p* = .022). The OCD group scored significantly higher on the Y‐BOCS, OCI‐R, BDI and STAI than the other two groups.

**TABLE 1:** Demographic and clinical data of participants

Characteristic	OCD (*n* = 46)	UFDR (*n* = 24)	HC (*n* = 49)	F/χ^2^	*p*
Demographic characteristic					
Age, years	25.72 ± 5.20	29.29 ± 7.77	26.06 ± 5.68	1.574^a^	.212
Gender (M/F)	33/13	12/12	27/22	4.136^b^	.126
Education	12.98 ± 2.84	12.96 ± 2.97	14.45 ± 2.73	3.942^a^	.022*
**Clinical characteristic**					
Y‐BOCS total	25.80 ± 5.07	0.88 ± 1.70	1.10 ± 2.14	694.710^a^	<.001^***^
Y‐BOCS obsessions	14.65 ± 3.19	0.38 ± 0.92	0.84 ± 1.68	521.396^a^	<.001^***^
Y‐BOCS compulsion	11.15 ± 4.96	0.50 ± 1.18	0.27 ± 0.73	164.134^a^	<.001^***^
OCI‐R	23.20 ± 12.63	8.50 ± 9.67	14.47 ± 12.63	12.972^a^	<.001^***^
BDI	17.59 ± 11.06	3.21 ± 5.77	7.51 ± 8.69	23.587^a^	<.001^***^
STAI state	51.39 ± 16.40	25.54 ± 17.97	35.16 ± 16.90	21.120^a^	<.001^***^
STAI trait	53.11 ± 14.57	25.58 ± 17.68	36.04 ± 17.21	25.531^a^	<.001^***^

*Note*: Scores are indicated as the mean ± *SD*.

Abbreviations: BDI, beck depression Inventory; HC, healthy control participants; OCD, obsessive–compulsive disorder patients; OCI‐R, obsessive compulsive inventory‐revised; STAI, state–trait anxiety inventory; UFDR, unaffected first‐degree relatives; Y‐BOCS, Yale‐Brown obsessive–compulsive scale.

^a^ One way ANOVA was used to compare across groups (OCD, UFDR, and HC).

^b^ Chi‐square test was used to compare categorical variables across groups (OCD, UFDR, and HC).

^*^ *p* < .05; ^***^ *p* < .001.

### 3.2 Intrinsic connectivity networks

Group ICA revealed 39 meaningful ICs. The spatial maps for each IC are shown in Figure 1, grouped into six networks based on overlap with established intrinsic connectivity networks (http://findlab.stanford.edu/functional_ROIs.html): auditory network (AUD: ICs 15, 71), visual network (VIS: ICs 31, 42, 49, 51, 54, 57, 69, 95), sensorimotor network (SMN: ICs 39, 44, 59, 62, 74, 77, 94), cognitive executive network (CEN: ICs 14, 25, 32, 35, 37, 43,75), default mode network (DMN: ICs 4, 12, 17, 33, 48, 53, 70, 87, 97, 98, 99), and cerebellum network (CB: ICs 10, 16, 84, 96). Detailed information about each independent component is given in Table S2.

## 4 dFNC STATE ANALYSES

### 4.1 Identification of states

**FIGURE 2:**
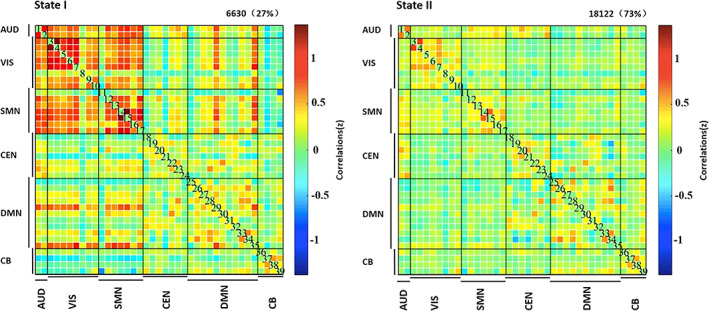
dFNC states identified across participants. For each state the dFNC matrix indicates the correlation between each pair of ICs. Refer to Figure 1 for spatial maps of each IC; the order of ICs within each network follows the order in Figure 1. The numbers at the top of the matrixes indicate the total number of windows characterized by the state and percentage of the total. Auditory network (AUD); visual network (VIS); sensorimotor network (SMN); cognitive executive network (CEN); default mode network (DMN); cerebellar (CB) network

Using the k‐means clustering algorithm, we identified two different functional connectivity states that were recurrent throughout the rs‐fMRI acquisition and across all participants, as shown in Figure 2. State I was less frequent (27% of the scan duration) characterized by substantial between‐network FC, particularly between ICs in the Auditory, Visual, Sensorimotor, and Default Mode networks. State II was more frequent (73% of scan duration) and was characterized by primarily within‐network functional connectivity and minimal between‐network functional connectivity.

### 4.2 Within state connectivity differences between groups

**FIGURE 3:**
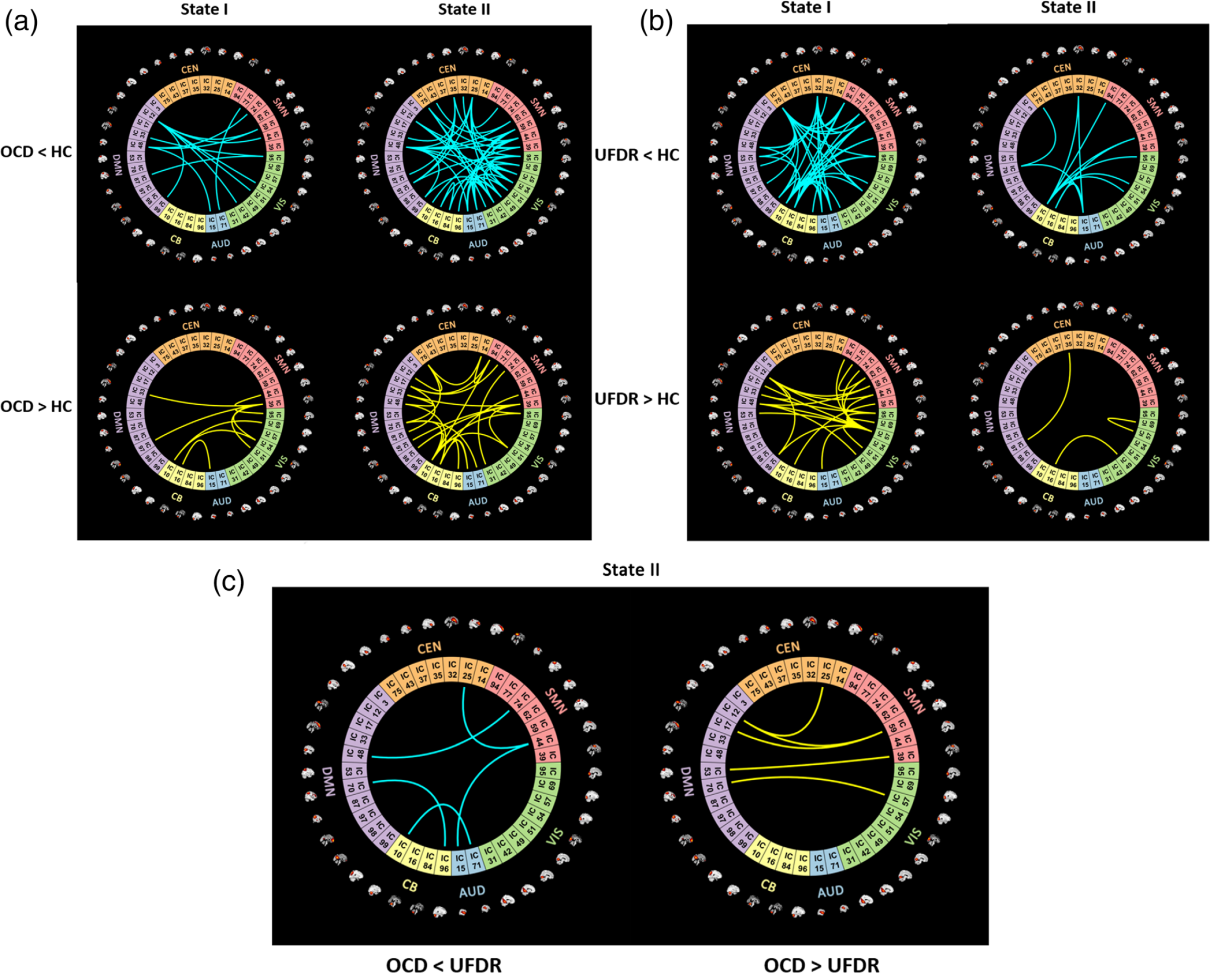
Functional connectivity differences in States I and II for the OCD, UFDR and HC groups. (a) Functional connectivity differences in States I and II between OCD and HC. The OCD group showed both weaker (cyan, top) and stronger (yellow, bottom) functional connectivity patterns in comparison to the HC group. (b) Functional connectivity differences in States I and II between UFDR and HC. (c) Functional connectivity differences in States II between OCD and UFDR. The color for each segment indicates which of the six networks the IC fell within. Blue: auditory network (AUD); Green: visual network (VIS); Red: sensorimotor network (SMN); Orange: cognitive executive network (CEN); Purple: default mode network (DMN); Yellow: cerebellar network (CB)

We compared the strength of connections across three participant groups at each state by ANOVA (Table S4), and the post hoc results are illustrated in Figures 3. For all comparisons we used a FDR correction and alpha of *p* < .05. When comparing OCD with HC, there were numerous differences in State II and relatively fewer in State I. In State II, we identified a total of 48 weaker connections in OCD compared to HC, including both within‐network (DMN, CB, VIS, SMN) and between‐network (DMN‐AUD, DMN‐VIS, DMN‐SMN, DMN‐CEN, AUD‐VIS, AUD‐SMN, VIS‐SMN, VIS‐CEN, SMN‐CEN, CB‐VIS, CB‐SMN) connections. We also observed 25 stronger connections in OCD than HC during State II, including both within‐network (DMN, CEN, CB) and between‐network (DMN‐SMN, DMN‐CB, DMN‐AUD, CEN‐SMN, SMN‐VIS, SMN‐CB, VIS‐CB, AUD‐CB). For State I we observed 12 weaker connections in OCD compared to HC, including within‐network (DMN) and between‐networks (DMN‐SMN, DMN‐VIS, DMN‐AUD, SMN‐AUD), and nine stronger connections, including within‐network (SMN) and between‐network (DMN‐SMN, SMN‐VIS, CB‐VIS, CB‐AUD) connections.

In contrast with the comparison with the OCD group, in which differences were predominantly found in State II, when the UFDR group was compared with the HC group the differences were predominantly in State I. In State I, we observed 40 weaker connections in UFDR compared to HC, including within‐network (CEN) and between‐networks (DMN‐CEN, DMN‐CB, CB‐AUD, CB‐VIS, CB‐SMN, AUD‐CEN, VIS‐CEN, SMN‐CEN). In addition, we observed that 22 stronger within‐network (VIS, SMN, DMN) and between‐network (DMN‐VIS, DMN‐SMN, CB‐SMN, VIS‐AUD, VIS‐SMN, SMN‐CEN) connections in UFDR compared to HC. For State II, we found that 12 weaker between‐network connections (AUD‐CEN, AUD‐DMN, AUD‐CB, VIS‐CB, CEN‐DMN, CEN‐CB, DMN‐CEN, CB‐SMN) in UFDR compared with HC while we observed that three stronger within‐network (VIS) connections and between‐network (DMN‐CEN, CB‐VIS) in UFDR compared with HC.

A direct comparison of the OCD and UFDR groups, revealed no significant differences in the strength of connections within State I. For State II, we found five weaker between‐network connections (AUD‐SMN, AUD‐CB, SMN‐CEN, SMN‐DMN, DMN‐SMN, DMN‐CB), and five stronger between‐network connections (DMN‐SMN, DMN‐CEN, DMN‐VIS) in OCD. Overall, these results indicate similar State I connectivity for OCD and UFDR that differs from State I connectivity in HC. In contrast, in State II UFDR were more similar to HC than to OCD: OCD differed from both HC and UFDR, whereas UFDR and HC showed relatively fewer differences when directly compared.

### 4.3 Temporal properties of the dynamic states

**FIGURE 4:**
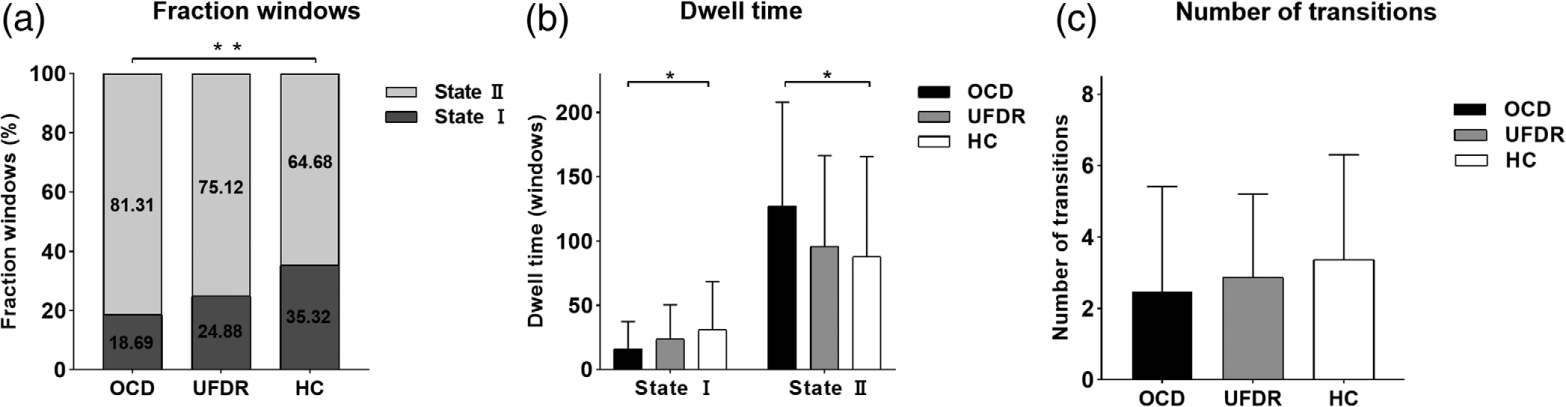
Differences in the temporal properties of per states for the OCD, UFDR, and HC groups. (a) The mean fractional windows indicating total percentage of spent in each state. (b) Mean dwell time, defined as number of consecutive windows spent in each state before switching. (c) Number of transitions, defined as total number of switches between states. Asterisks indicate a significant group difference (* *p* < .05, ** *p* < .01)

We examined the proportion of time spent in each of the two states and frequency of switching to and from State I and State II. There was a significant group difference in the fraction of total time spent in each of the two states (Figure 4a) (F_[2,116]_ = 3.927, *p* = .022). A post hoc test found that State I was less frequent in OCD (18.69 ± 24.63%) than in HC (35.32 ± 32.78%) (*p*<.01) while State II was more frequent in OCD (81.31 ± 24.63%) than in HC (75.12 ± 29.25%) (*p*<.01). The UFDR showed numerically intermediate values that did not differ significantly from either the OCD or HC group. There were also significant differences in dwell time (mean time spent in a state before switching to the other state), as illustrated in Figure 4b. There was a significant across‐groups effect for State I mean dwell time (F_[2,116]_ = 3.077, *p* = .05); post hoc tests revealed that OCD spent significantly less time in State I before switching to State II than HC (*p*<.05). There was also a significant across‐group effect for State II mean dwell time (F_[2,116]_ = 3.168, *p* = .046), such that OCD spent longer in State II before switching to State I than HC. Again, UFDR showed numerically intermediate values between OCD and HC groups. The three groups did not differ significantly in the total number of transitions between states, (F_[2,116]_ = 1.177, *p* = .312) (Figure 4c).

### 4.4 Correlation between clinical measures and dFNC temporal properties

**FIGURE 5:**
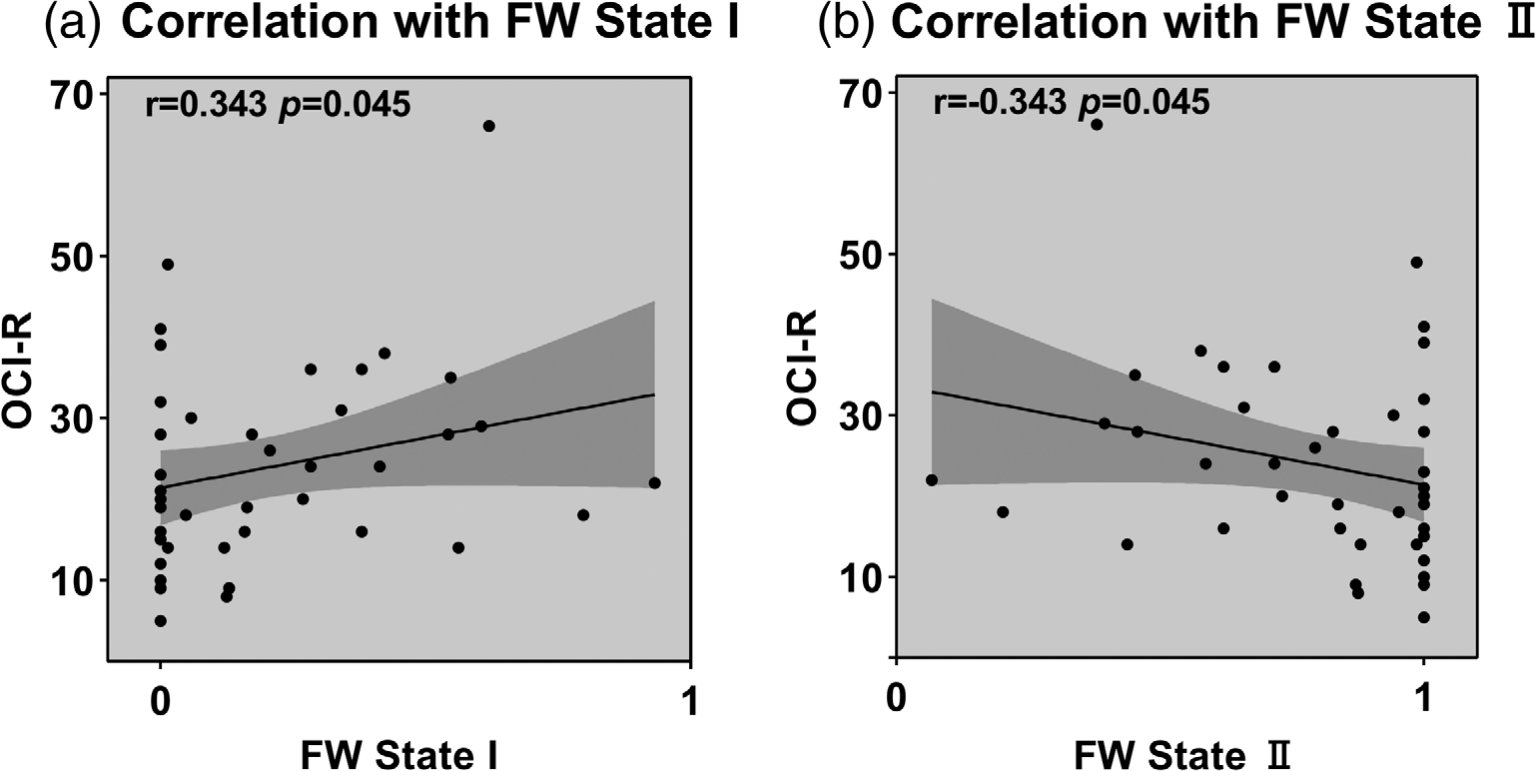
Correlation between OCD symptoms (measured via the OCI‐R) and the temporal properties for the OCD group. (a) Proportion of time (measured as fractional windows) in State I positively correlated with OCI‐R score. (b) Proportion of time (measured as fractional windows) in State II negatively correlated with the OCI‐R score

We examined correlations between dFNC properties and clinical characteristics in the OCD group, controlling for both BDI and STAI scores and education level. The proportion of time spent in State I (fractional windows) was positively correlated with OCI‐R scores (Figure 5a; *r* = .343, *p*<.05) while proportion of time spent in State II was negatively correlated with OCI‐R scores (Figure 5b; *r* = −.343, *p*<.05, FDR correction). A full reporting of all clinical measure correlations is provided in Table S3.

## 5 DISCUSSION

This is the first study using dynamic functional network connectivity that compared obsessive–compulsive disorder patients with both their first‐degree relatives and healthy controls. We identified two distinct connectivity states that were present across all three groups. State I was a less frequent (27%), integrated state characterized by the predominance of between‐network connections, and State II was a more frequent (73%), segregated state characterized by within‐network connections. OCD patients differed from HC in both temporal features and within‐State patterns of network connectivity. For temporal features, OCD patients spent more time in State II, and less time in State I, than healthy controls. Clinical features were correlated with more time in State II and less time in State I. Together these results indicate that OCD may be associated with more tendency to get “stuck” in the highly modular State II. The UFDR group showed intermediate values between OCD patients and HC, indicating that the temporal dynamics of functional connectivity could be a useful biomarker. When within‐State connectivity was examined, OCD differed from HC in many ways in both State I and State II. However, the UFDR showed a more OCD‐like pattern in State I, and a more HC‐like pattern in State II, indicating that State I connectivity might also serve as a biomarker.

### 5.1 Network connectivity within states

Our study provides additional insight into cortico‐cortico network differences in OCD. Many previous studies focused on CSTC networks with the striatum as the core (Alexander, DeLong, & Strick, 1986). However, there is growing evidence that people with OCD have a wider range of brain network disorders (Fan et al., 2017b; Hou et al., 2012) We found within State I, generally characterized by between ‐network connectivity, that network connectivity between the DMN and other networks (SMN, VIS) was lower in OCD. The DMN has been related to the brain's monitoring of internal and external environment, emotional processing, creativity, self‐reflection, and episodic memory extraction (Raichle, 2015). Our results are consistent with previous studies that also found lowered default network in functional activity and connectivity in OCD (Peng et al., 2014; Shin et al., 2014). The influential “triple network model” in OCD (Anticevic et al., 2014; Beucke et al., 2013; Harrison et al., 2009) proposes that DMN is modulated by the salience network. Our results were also consistent with Kwak and colleagues who indicated that not only within‐DMN rs‐FC but also functional connectivity between brain regions involved in the DMN were critical and that rs‐FC features in somatosensory‐motor, visual and auditory, and cingulo‐opercular networks were associated with clinical symptom severity improvement. (Kwak et al., 2020). We also found within State I that connectivity within motor and sensory networks (CB‐AUD, CB‐VIS, SMN‐VIS) was greater in OCD compared with HC. The cerebellum plays an important role in cognition and emotion, in addition to motor function (D'Angelo & Casali, 2012). Previous research has associated OCD with abnormal cerebellar structure and function. At rest, OCD patients show lower spontaneous activity of the cerebellum (Hou et al., 2014), and the functional connection strength between cerebellum and the whole brain increases (Anticevic et al., 2014).

Within State II, OCD showed reduced between‐network connectivity and increased within‐network connectivity. This overall higher modularity is consistent with previous studies. Zhang et al. (2011) characterized networks using graph theory and found that OCD had abnormally higher clustering coefficient and shortest path length, both consistent with high modularity and network segregation. In contrast networks in HC controls were characterized by the small‐world property, which has been shown to indicate an effective balance between modularization and decentralized information processing.

### 5.2 Temporal dynamics of state transitions

We found that patients with OCD spent longer in State II, both overall (fraction window) and for each individual instance (dwell time), and less time in State I, than the HC group. These results reveal that OCD patients were engaged across time in a brain configuration pattern characterized by a lack of between‐network connections at the expense of strong within‐network connections. Although there was no significant difference in number of transitions among the three groups, there was a trend for OCD to have fewer transitions than HC. These results are consistent with a study of schizophrenia and ASD, which found that both clinical groups displayed an increased time spent in a state of weak, intra‐network connectivity (Rabany et al., 2019). In contrast, a study of Parkinson disease found that these patients showed more time in a State characterized by between‐network connections (Kim et al., 2017). In addition, the temporal properties were significantly associated with clinical features. The proportion of time in State I (fractional windows) was positively correlated with OCI‐R scores and proportion of time in State II was negatively correlated with OCI‐R scores, indicating that those with higher OCD severity spend more time in the highly segregated state. These findings emphasized the importance of dFNC studies, as it could reveal potential characteristic of OCD.

One previous study examined temporal properties of dFNC in OCD (Liu et al., 2020). It is hard to directly compare the studies because the data‐driven algorithm extracted four different states in the Liu study between which the OCD group showed more frequent switching than controls, whereas our study the algorithm indicated two states with no significant difference in number of transitions. The two studies also differed in that Liu et al examined treatment naive unmedicated participants whereas in our study participants were receiving medication therapy. Future research will be needed to determine how these varying factors may affect functional connectivity in OCD.

### 5.3 dFNC as an endophenotype for OCD

Our results show a number of similarities between OCD and their UFDR which may prove useful as a biomarker reflecting a shared endophenotype for OCD and UFDR. Previous studies have shown that dFNC may reflect various aspects of the neural system functional capacity (Deco, Jirsa, & McIntosh, 2011; Kucyi et al., 2016) and thus, may serve as a novel physiological biomarker of disease (Damaraju et al., 2014; Hutchison et al., 2013). A number of studies have identified shared features between OCD and their UFDR. For instance, a meta‐analysis of OCD suggested that abnormalities in inhibition, planning/problem solving, and reward‐based decision‐making are shared features of OCD and their UFDR and might be trait markers related to vulnerability for developing OCD (Bora, 2020). Dong and colleagues found that the same changes in effective connectivity were present in both OCD patients and their unaffected first‐degree relatives (Dong et al., 2020).

With regard to the temporal properties, although there were no significantly differences between UFDR and the HC and OCD groups, the UFDR group had numerically intermediate values on all the measures. This pattern suggestion that dynamic functional network connectivity alterations can be considered, at least partly, an endophenotype of OCD. This result was similar to the previous study (Fan et al., 2016). With regard to within‐state connectivity patterns we found evidence that State I connectivity may be an especially promising candidate to use as a biomarker of OCD. Overall OCD and UFDR showed greatest similarity in State I: there were no significant differences in connectivity between the groups in State I, whereas both OCD and UFDR showed multiple differences in connectivity when compared with HC. In contrast, in State II UFDR appeared to be more similar to HC than OCD. State I was the less frequent and highly integrated state, whereas State II was the more highly modular state. Overall, then, UFDR show OCD like patterns of between‐network integration in State I, but HC like patterns of modularity in State II.

## 6 LIMITATIONS

Several limitations should be taken into account for the current study. First, OCD patients were being treated with various types of therapy and medication. Therefore, it is possible that the results were influenced by these treatment conditions. However, we found that there were no significantly different in temporal properties of dFNC between medicated and unmedicated OCD patients (Table S5). Second, the sample size of UFDR was limited. Third, the relatively small sample size in this study and clinical heterogeneity of comorbidity across OCD patients, may have contributed variance to the study. Future studies with larger sample size, OCD subgroups and drug‐naive patients are needed to confirm our results. Fourth, the HC group scored higher on STAI than UFDR. This may be due to the fact that many of the controls were college students who participated in the experiment during the final exam period, thus leading to their high anxiety scores.

## 7 CONCLUSION

We identified several dFNC differences in OCD and UFDR that may be useful for establishing biomarkers of an OCD endophenotype. First, OCD patients had abnormal temporal properties which correlated with clinical features, which were shared with their UFDR. Second, we found similar connectivity patterns for UFDR and OCD within a dFNC state characterized by between‐network integration. These results provide new insights into the pathophysiology of OCD patients and indicate dFNC measures that could be used as biomarker to identify those at risk.

## Supporting information

**Appendix** S1: Supporting Information

## Data Availability

The original data are available from the first author on request.
