# Supplementary material for: Abnormal brain functional network dynamics in obsessive–compulsive disorder patients and their unaffected first‐degree relatives
Source: Hum Brain Mapp. 2021 Jun 5;42(13):4387–98. doi: 10.1002/hbm.25555 (PMC8356985; doi:10.1002/hbm.25555)
Supplement: Supplementary file 1 — Appendix S1: Supporting Information [file HBM-42-4387-s001.docx]

**Abnormal Brain Functional Network Dynamics in Obsessive-Compulsive Disorder Patients and Their Unaffected First-Degree Relatives.**

*Supplementary Material*

**Supplementary Table S1** Treatment details of OCD patients.

| **Treatment** | **Number of case** | **Average dosage (mg)** | |
| --- | --- | --- | --- |
| citalopram | 3 | | 40 |
| clomipramine | 2 | | 100 |
| escitalopram | 3 | | 20 |
| fluoxetine | 2 | | 40 |
| mirtazapine | 3 | | 30 |
| paroxetine | 3 | | 40 |
| sertraline | 5 | | 150 |
| paroxetine + sodium valproate | 2 | | 40 + 500 |
| paroxetine + quetiapine | 3 | | 40 + 200 |

**Supplementary Table S2** Peak activation information of 39 independent components

| **ICN Regions** | | **BA** | ***k*** | ***t*_max_** | **Peak coordinate (mm)** | | |
| --- | --- | --- | --- | --- | --- | --- | --- |
|  |  |  |  |  | **x** | **y** | **z** |
|  | **Auditory Network (AUD)** | | | | | | |
| IC15 R Superior Temporal Gyrus  L Superior Temporal Gyrus | | 22 | 1473  1664 | 31.06  35.18 | 57  -57 | 3  0 | -6  -3 |
| IC71 R Superior Temporal Gyrus | | 41 | 1947 | 32.38 | 57 | -18 | 9 |
| L Superior Temporal Gyrus | |  | 2127 | 36.52 | -48 | -21 | 12 |
|  | **Sensorimotor Network (SMN)** | | | | | | |
| IC39 Medial Frontal Gyrus  IC44 Medial Frontal Gyrus | | 6  6 | 6613  3099 | 33.50  32.12 | -6  -3 | -12  -15 | 57  69 |
| IC59 Postcentral Gyrus | | 2 | 3820 | 31.58 | 21 | -45 | 60 |
| IC62 L Postcentral Gyrus | | 2 | 2839 | 27.07 | -48 | -27 | 48 |
| IC74 R Postcentral Gyrus | | 3 | 2777 | 28.95 | 45 | -30 | 51 |
| IC77 L Precentral Gyrus  R Precentral Gyrus | | 43  43 | 1901  1650 | 30.55  30.64 | -54  54 | -6  -9 | 30  24 |
| IC94 Paracentral lobule | | 6 | 3777 | 37.13 | -6 | -33 | 60 |
|  | **Visual Network (VIS)** | | | | | | |
| IC31 R Lingual Gyrus | | 19 | 4124 | 33.36 | 12 | -48 | 0 |
| IC42 L Lingual Gyrus | | 30 | 4121 | 32.57 | -18 | -51 | 0 |
| IC49 Lingual Gyrus | | 18 | 4698 | 34.71 | 6 | -75 | -9 |
| IC51 R Calcarine Gyrus | | 17 | 3536 | 38.99 | 15 | -63 | 6 |
| IC54 Bi cuneus | | 31 | 3506 | 34.36 | 9 | -72 | 18 |
| IC57 Bi Middle Occipital Gyrus | | 18 | 4842 | 27.86 | 30 | -87 | 3 |
| IC69 Bi Lingual Gyrus | | 17 | 2933 | 26.15 | 24 | -87 | -9 |
| IC95 L Calcarine Gyrus | | 18 | 2775 | 32.06 | 0 | -90 | 3 |
|  | **Cognitive Executive Network (CEN)** | | | | | | |
| IC14 R Superior Frontal Gyrus | | 10 | 3283 | 25.95 | 27 | 60 | 6 |
| IC25 Inferior Frontal Gyrus  L Inferior Parietal Lobe | | 47  40 | 2289  1206 | 30.24  21.00 | -45  -57 | 36  -36 | 9  39 |
| IC32 Middle Frontal Gyrus | | 32 | 3558 | 34.44 | -6 | 30 | 18 |
| IC35 Inferior Frontal Gyrus | | 47 | 1340 | 34.47 | -42 | 15 | -3 |
| IC37 L Inferior Frontal Gyrus  R Inferior Frontal Gyrus | | 9  45 | 2493  2260 | 36.73  27.32 | -48  51 | 15  24 | 27  24 |
| IC43 Inferior Frontal Gyrus | | 47 | 1994 | 37.77 | -48 | 27 | -12 |
| IC75 L Superior Temporal Gyrus  R Insular Gyrus | | 38  21 | 1394  606 | 31.00  25.70 | -39  42 | 0  -3 | -12  -9 |
|  | **Default Mode Network (DMN)** | | | | | | |
| IC4 L inferior orbitofrontal gyrus  L angular gyrus  L Precuneus | | 47  40  7 | 1806  1351  666 | 40.71  28.76  13.59 | -45  -45  -6 | 42  -63  -66 | -9  39  30 |
| IC12 R angular gyrus  L medial orbitofrontal gyrus  R middle frontal gyrus | | 39  19  8 | 2157  995  951 | 32.73  28.78  20.74 | 48  -36  27 | -57  -69  24 | 33  39  45 |
| IC17 L Precuneus | | 7 | 3689 | 39.11 | -3 | -54 | 39 |
| IC33 medial superior frontal gyrus | | 9 | 3046 | 34.06 | 0 | 42 | 45 |
| IC48 L middle temporal gyrus  R middle temporal gyrus | | 39  21 | 1496  1892 | 27.25  29.20 | -54  51 | -57  -60 | 9  6 |
| IC53 L superior parietal gyrus  R inferior parietal gyrus | | 40  40 | 2270  663 | 28.68  20.12 | -30  45 | -66  -48 | 48  51 |
| IC70 L inferior parietal gyrus  R inferior parietal gyrus  R inferior frontal gyrus | | 40  7  9 | 522  2264  1749 | 15.71  29.56  17.91 | -39  48  51 | -57  -42  15 | 51  45  24 |
| IC87 L supramarginal gyrus  R supramarginal gyrus | | 40  40 | 1699  2086 | 31.15  30.93 | -57  57 | -48  -42 | 33  27 |
| IC97 L medial orbitofrontal gyrus | | 10 | 2106 | 31.19 | -6 | 51 | -6 |
| IC98 R Precuneus | | 31 | 2523 | 43.77 | 6 | -57 | 27 |
| IC99 R superior occipital gyrus | | 7 | 4031 | 32.23 | 24 | -66 | 42 |
|  | **Cerebellum Network** | | | | | | |
| IC10 R Cerebellum | | - | 2750 | 28.61 | 36 | -63 | -30 |
| IC16 L Cerebellum | | - | 2807 | 35.84 | -3 | -63 | -30 |
| IC84 R Cerebellum | | - | 2221 | 35.11 | 9 | -66 | -51 |
| IC96 R Cerebellum | | - | 2593 | 36.41 | 12 | -84 | -30 |

Abbreviations: IC, independent component; BA, Brodmann Area; k, the cluster size; L, left; R, right

*Group-discriminating dFNC Features with Different Window Sizes*

We used a sliding window approach with different window sizes (range from 18 TRs to 26 TRs: 36 s to 52 s) to analyze the effect of different window lengths on the results. Similar to the analysis in the main manuscript, the number of clusters was determined as k = 2, which was consistent with that in our manuscript. We also used three different indices to examine the temporal properties of the DFNC states: (1) Fractional window (FW) defined as the proportion of time spent in each state; (2) mean dwell time (DT) defined as the mean length of time the participant remained in each state before switching to another state (3) number of transitions, defined as the total number of changes between states the participant made across the entire resting state scan. Group differences were assessed using ANOVA, applying a least significant difference (LSD) post-hoc test.

The cluster centroids and the group comparison results of dFNC features with different window sizes are displayed in Figure S3 ~ Figure S6. The overall results are highly consistent across different window sizes and similar to the results presented in the main manuscript. On one hand, the identified reoccurring dFNC states and their connectivity patterns are highly similar across different window sizes. On the other hand, OCD patients show consistently alterations in fraction rate, mean dwell time and transitions of dFNC states captured by the sliding window approach with different window sizes. OCD patients spent more time in State II (a more frequent, segregated state with strong within-network connections) and less time in State I (a less frequent, integrated state characterized by the predominance of between-network connections) than HC, as measured by fractional windows and mean dwell time. Time in each state for the UFDR were intermediate between OCD patients and HC.

**Supplementary Figure S1** The group-discriminating fraction rate of states with window size of 18 TR. Upper: the cluster centroids of four functional network connectivity (dFNC) states. Lower: group comparisons in temporal properties of two dFNC states. Significant group difference is indicated by asterisks.


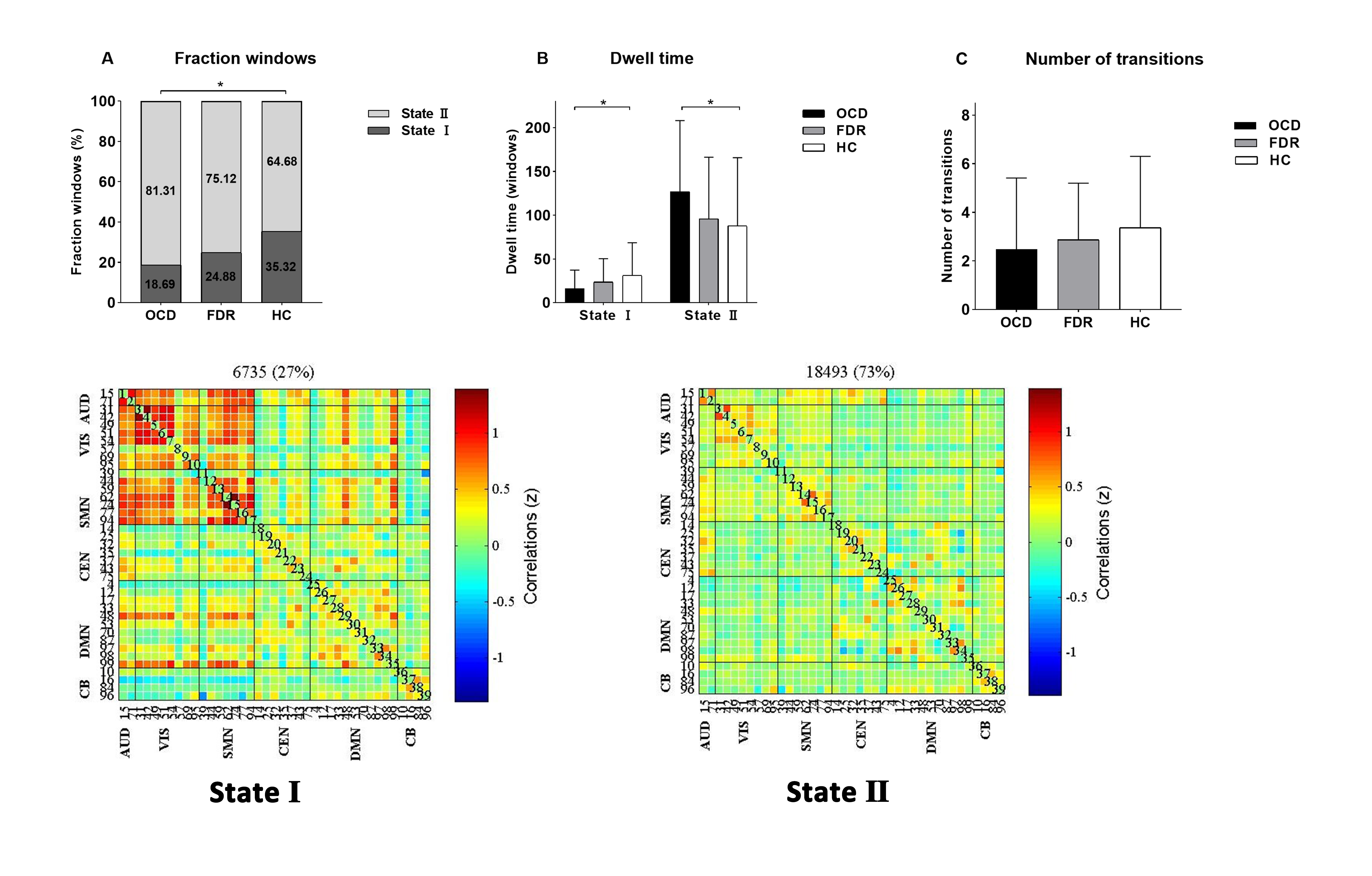


**Supplementary Figure S2** The group-discriminating fraction rate of states with window size of 20 TR. Upper: the cluster centroids of four functional network connectivity (dFNC) states. Lower: group comparisons in temporal properties of two dFNC states. Significant group difference is indicated by asterisks e (* *p* < 0.05, ** *p* < 0.01).


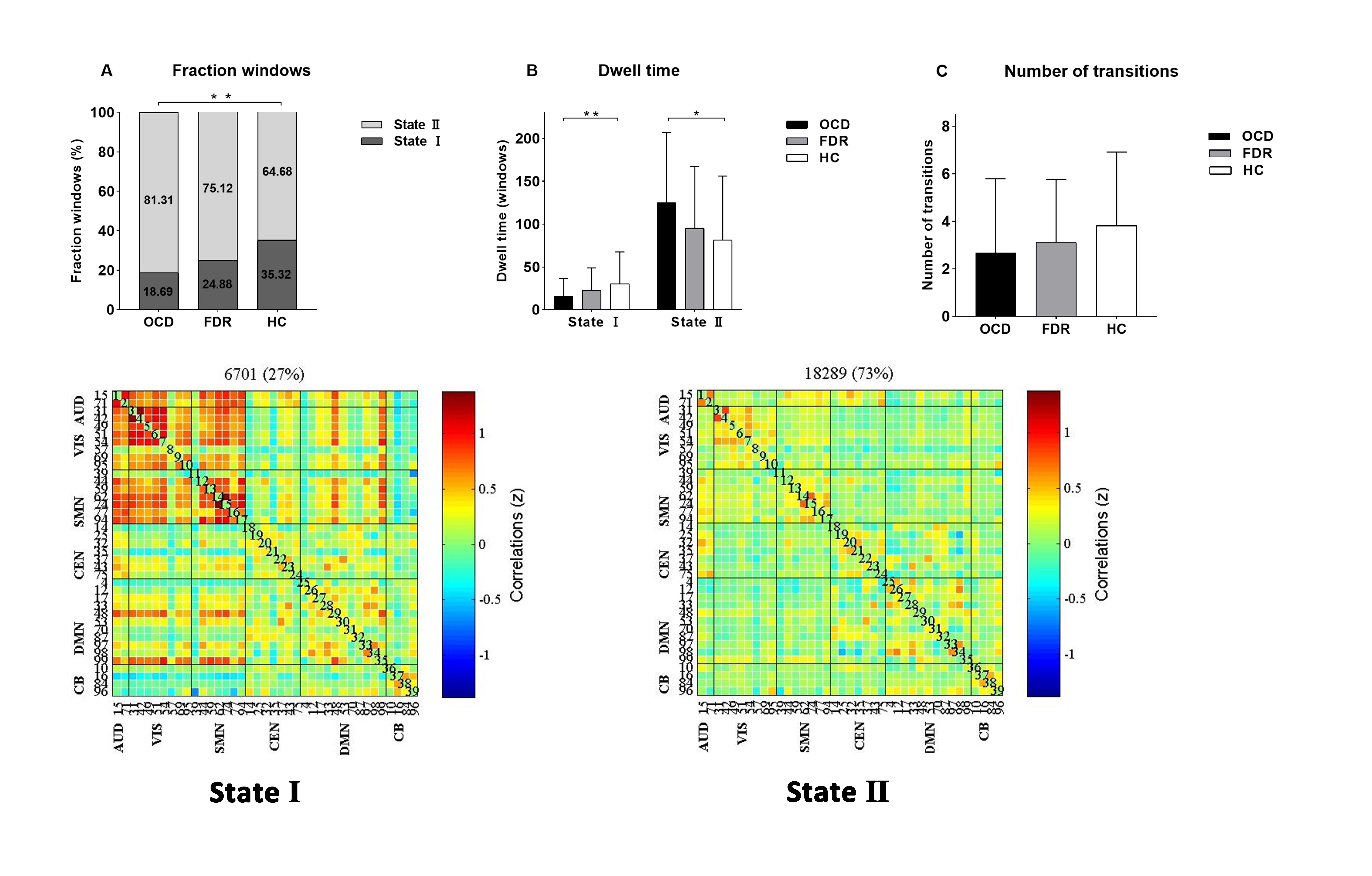


**Supplementary Figure S3** The group-discriminating fraction rate of states with window size of 24 TR. Upper: the cluster centroids of four functional network connectivity (dFNC) states. Lower: group comparisons in temporal properties of two dFNC states. Significant group difference is indicated by asterisks e (* *p* < 0.05, ** *p* < 0.01).


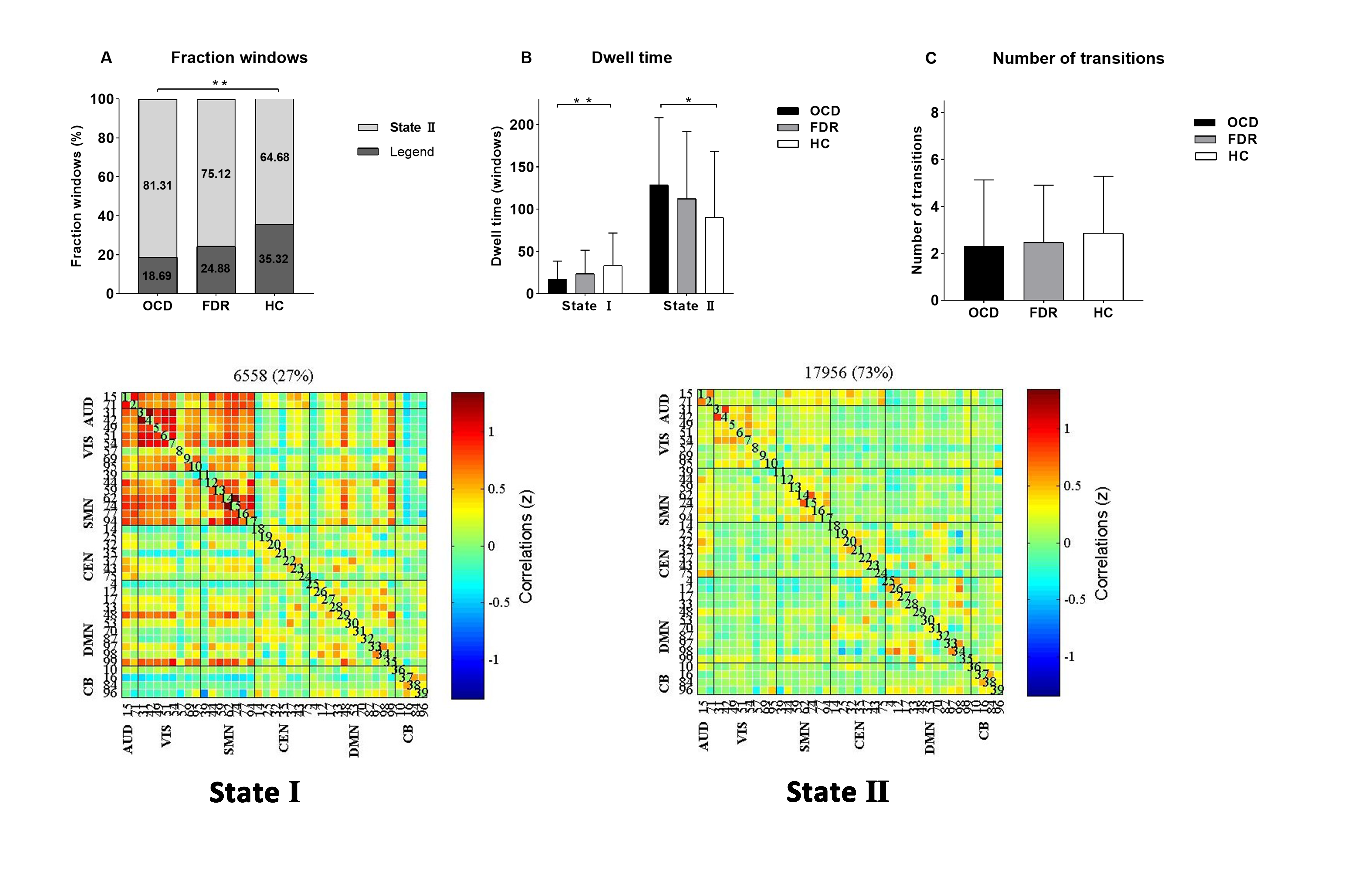


**Supplementary Figure S4** The group-discriminating fraction rate of states with window size of 26 TR. Upper: the cluster centroids of four functional network connectivity (dFNC) states. Lower: group comparisons in temporal properties of two dFNC states. Significant group difference is indicated by asterisks e (* *p* < 0.05, ** *p* < 0.01).


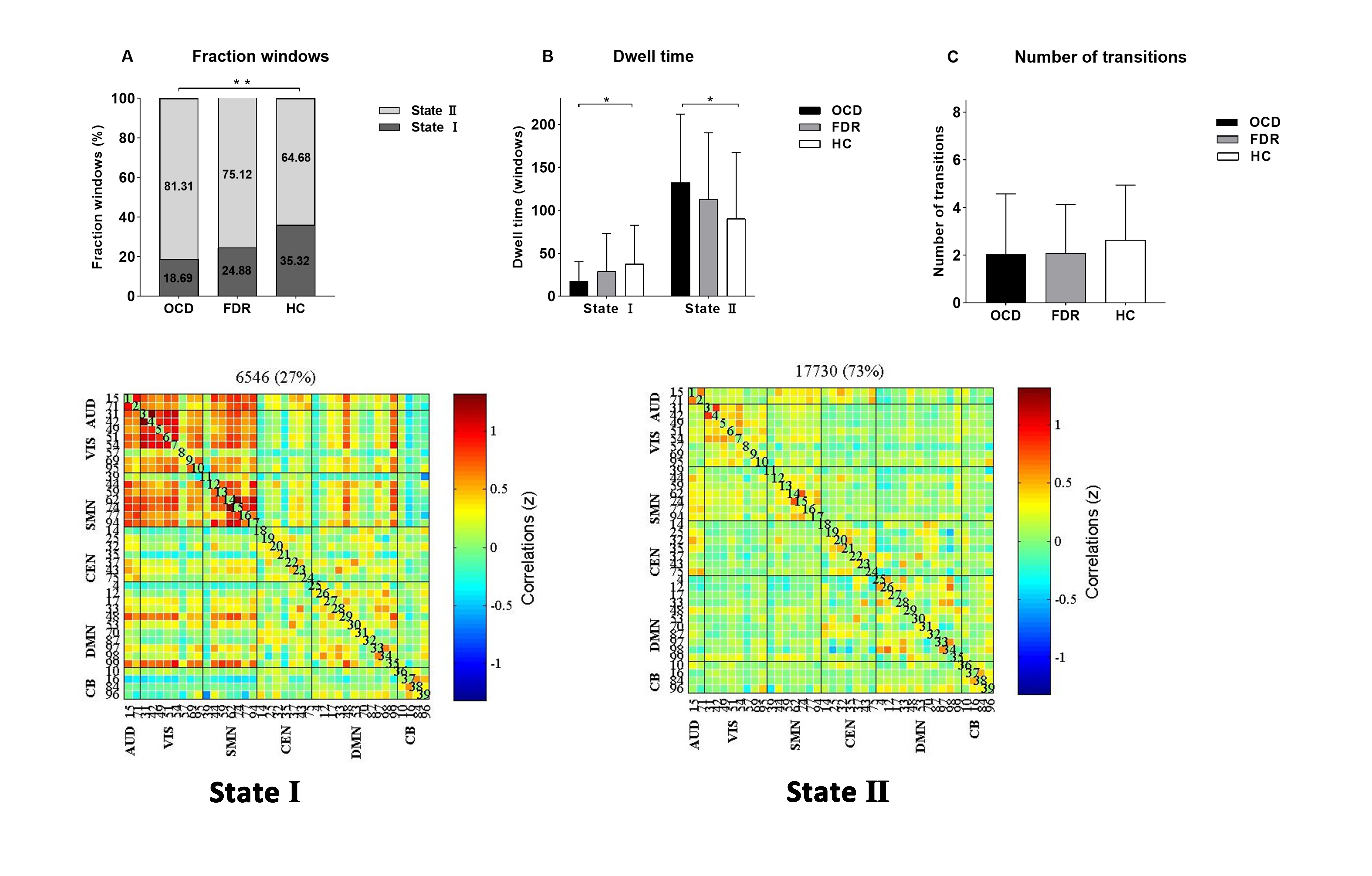


**Supplementary Table S3** Correlation of the clinical scores with the temporal properties for the OCD group. BDI, STAI and education were controlled (*p*<0.05, FDR correction).

|  | OCI-R | YBOCS |
| --- | --- | --- |
| S1FW | r=0.343  *p*=0.045 | r=0.296  *p*=0.135 |
| S2FW | r=-0.343  *p*=0.045 | r=-0.296  *p*=0.135 |
| S1DT | r=0.131  *p*=0.402 | r=0.171  *p*=0.301 |
| S2DT | r=-0.259  *p*=0.117 | r=-0.161  *p*=0.301 |
| Transition | r=0.337  *p*=0.045 | r=0.178  *p*=0.301 |

**Supplementary Table S4a** ANOVA results at State Ⅰ.

| **ICs** | **F** | ***p*** |
| --- | --- | --- |
| 57-15 | 8.25 | <0.001 |
| 95-57 | 8.65 | <0.001 |
| 39-51 | 13.97 | <0.001 |
| 39-54 | 11.11 | <0.001 |
| 39-95 | 12.57 | <0.001 |
| 44-57 | 7.42 | 0.0012 |
| 44-39 | 10.58 | <0.001 |
| 62-57 | 6.41 | 0.0028 |
| 62-39 | 8.60 | <0.001 |
| 74-57 | 7.40 | 0.0012 |
| 77-71 | 6.15 | 0.0034 |
| 77-62 | 6.55 | 0.0024 |
| 94-57 | 6.06 | 0.0037 |
| 32-15 | 8.53 | <0.001 |
| 32-71 | 8.91 | <0.001 |
| 32-62 | 6.10 | 0.0036 |
| 32-74 | 7.18 | 0.0014 |
| 32-94 | 7.09 | 0.0016 |
| 43-57 | 6.67 | 0.0022 |
| 75-15 | 7.08 | 0.0016 |
| 75-71 | 7.00 | 0.0017 |
| 75-51 | 8.87 | <0.001 |
| 75-54 | 6.43 | 0.0027 |
| 75-95 | 7.41 | 0.0012 |
| 75-62 | 7.27 | 0.0013 |
| 75-74 | 6.74 | 0.0021 |
| 75-94 | 7.49 | 0.0011 |
| 75-25 | 6.27 | 0.0031 |
| 12-42 | 7.44 | 0.0011 |
| 12-51 | 7.82 | <0.001 |
| 12-54 | 7.56 | <0.001 |
| 12-95 | 6.91 | 0.0018 |
| 12-44 | 8.90 | <0.001 |
| 12-59 | 10.55 | <0.001 |
| 12-94 | 6.40 | 0.0028 |
| 17-57 | 11.68 | <0.001 |
| 33-95 | 11.42 | <0.001 |
| 33-39 | 7.01 | 0.0012 |
| 33-12 | 8.33 | <0.001 |
| 48-15 | 6.57 | 0.0024 |
| 48-57 | 8.35 | <0.001 |
| 48-59 | 9.83 | <0.001 |
| 48-74 | 6.93 | 0.0018 |
| 48-32 | 6.12 | 0.0035 |
| 48-75 | 7.12 | 0.0015 |
| 53-57 | 5.90 | 0.0043 |
| 53-39 | 7.10 | 0.0015 |
| 53-32 | 5.74 | 0.0049 |
| 53-75 | 6.82 | 0.0019 |
| 70-14 | 5.74 | 0.0049 |
| 70-75 | 7.63 | <0.001 |
| 87-95 | 6.24 | 0.0032 |
| 97-39 | 6.85 | 0.0019 |
| 97-12 | 5.82 | 0.0045 |
| 98-39 | 7.01 | 0.0017 |
| 99-57 | 6.60 | 0.0024 |
| 99-75 | 7.03 | 0.0016 |
| 99-12 | 9.01 | <0.001 |
| 10-15 | 8.59 | <0.001 |
| 10-71 | 6.42 | 0.0027 |
| 10-31 | 7.36 | 0.0012 |
| 10-51 | 8.56 | <0.001 |
| 10-54 | 10.62 | <0.001 |
| 10-69 | 7.83 | <0.001 |
| 10-95 | 7.12 | 0.0015 |
| 10-44 | 6.43 | 0.0027 |
| 10-62 | 5.95 | 0.0041 |
| 10-94 | 6.61 | 0.0023 |
| 10-48 | 10.11 | <0.001 |
| 10-99 | 7.04 | 0.0016 |
| 16-15 | 10.98 | <0.001 |
| 16-95 | 9.33 | <0.001 |
| 16-39 | 9.45 | <0.001 |
| 16-44 | 7.09 | 0.0016 |
| 16-59 | 6.00 | 0.0039 |
| 16-62 | 6.42 | 0.0027 |
| 16-74 | 6.45 | 0.0027 |
| 16-94 | 6.32 | 0.0030 |
| 16-48 | 5.77 | 0.0048 |
| 96-62 | 7.01 | 0.0017 |

**Supplementary Table S4b** ANOVA results at State Ⅱ.

| **ICs** | **F** | ***p*** |
| --- | --- | --- |
| 42-15 | 5.58 | 0.0049 |
| 42-71 | 12.49 | <0.001 |
| 49-71 | 6.82 | 0.0016 |
| 54-15 | 8.34 | <0.001 |
| 57-49 | 7.16 | 0.0012 |
| 57-54 | 5.66 | 0.0045 |
| 69-57 | 15.26 | <0.001 |
| 95-51 | 10.65 | <0.001 |
| 95-54 | 7.98 | <0.001 |
| 39-51 | 6.59 | <0.001 |
| 39-95 | 11.64 | <0.001 |
| 44-15 | 10.32 | <0.001 |
| 44-95 | 6.70 | 0.0018 |
| 44-39 | 5.90 | <0.001 |
| 59-15 | 5.61 | 0.0047 |
| 59-71 | 5.78 | 0.0040 |
| 59-39 | 6.00 | 0.0033 |
| 59-44 | 13.00 | <0.001 |
| 14-94 | 7.67 | <0.001 |
| 25-49 | 5.87 | 0.0037 |
| 25-44 | 13.23 | <0.001 |
| 25-59 | 5.65 | 0.0046 |
| 32-15 | 10.95 | <0.001 |
| 32-54 | 8.93 | <0.001 |
| 35-44 | 9.55 | <0.001 |
| 43-62 | 6.82 | 0.0016 |
| 75-42 | 6.17 | 0.0029 |
| 75-51 | 5.91 | 0.0036 |
| 75-14 | 7.63 | <0.001 |
| 12-51 | 5.92 | 0.0036 |
| 12-95 | 8.85 | <0.001 |
| 12-59 | 10.41 | <0.001 |
| 12-25 | 6.86 | 0.0015 |
| 12-75 | 10.88 | <0.001 |
| 17-59 | 6.12 | 0.0030 |
| 17-94 | 6.23 | 0.0027 |
| 17-12 | 5.87 | 0.0038 |
| 33-35 | 6.60 | 0.0019 |
| 48-15 | 7.20 | 0.0011 |
| 48-71 | 5.61 | 0.0048 |
| 53-39 | 7.31 | <0.001 |
| 53-74 | 5.55 | 0.0050 |
| 70-15 | 6.48 | 0.0021 |
| 70-71 | 6.04 | 0.0032 |
| 70-57 | 6.87 | 0.0015 |
| 70-75 | 8.54 | <0.001 |
| 87-17 | 5.67 | 0.0014 |
| 97-95 | 8.29 | <0.001 |
| 97-39 | 6.19 | 0.0028 |
| 97-25 | 8.94 | <0.001 |
| 97-35 | 9.29 | <0.001 |
| 99-57 | 6.01 | 0.0033 |
| 99-75 | 6.01 | 0.0033 |
| 10-15 | 13.03 | <0.001 |
| 10-71 | 8.47 | <0.001 |
| 10-51 | 11.84 | <0.001 |
| 10-54 | 7.07 | <0.001 |
| 10-95 | 10.28 | <0.001 |
| 10-39 | 11.29 | <0.001 |
| 10-44 | 7.31 | <0.001 |
| 10-94 | 9.94 | <0.001 |
| 10-32 | 7.26 | <0.001 |
| 10-70 | 6.96 | 0.0014 |
| 10-98 | 6.08 | 0.0031 |
| 16-15 | 8.36 | <0.001 |
| 16-49 | 8.53 | <0.001 |
| 16-44 | 10.88 | <0.001 |
| 84-69 | 6.99 | 0.0014 |
| 84-16 | 8.24 | <0.001 |
| 96-51 | 9.15 | <0.001 |
| 96-70 | 6.88 | 0.0015 |

**Supplementary Table S5** Differences in the temporal properties of per states for the medicated OCD, unmedicated OCD and HC groups.

|  | **Medicated(Med)** | **Unmedicated(unM)** | **HC** | **F** | ***p*** | **Post-hoc** |
| --- | --- | --- | --- | --- | --- | --- |
| **S1FW** | 0.20±0.28 | 0.17±0.20 | 0.35±0.33 | 3.903 | 0.024 | HC>Med, *p*=0.035  HC>unM, *p*=0.019  Med>unM, *p*=0.710 |
| **S2FW** | 0.80±0.28 | 0.83±0.20 | 0.65±0.33 | 3.903 | 0.024 | HC＜Med, *p*=0.035  HC＜unM, *p*=0.019  Med<unM, *p*=0.710 |
| **S1DT** | 14.07±21.27 | 18.51±21.43 | 31.23±37.36 | 3.028 | 0.053 | HC>Med, *p*=0.024  HC>unM, *p*=0.122  Med<unM, *p*=0.629 |
| **S2DT** | 124.65±86.91 | 129.70±75.21 | 87.86±77.92 | 2.845 | 0.063 | HC＜Med, *p*=0.061  HC＜unM, *p*=0.052  Med<unM, *p*=0.832 |
| **Transition** | 2.88±3.44 | 1.95±2.06 | 3.37±2.94 | 1.661 | 0.196 |  |
